# Genetically Determined Serum 25-Hydroxyvitamin D Is Associated with Total, Trunk, and Arm Fat-Free Mass: A Mendelian Randomization Study

**DOI:** 10.1007/s12603-021-1696-1

**Published:** 2021-11-12

**Authors:** Richard Kirwan, M. Isanejad, I.G. Davies, M. Mazidi

**Affiliations:** 1 School of Biological and Environmental Sciences, Liverpool John Moores University, Liverpool, UK; 2 Department of Musculoskeletal and Ageing Science, University of Liverpool, Liverpool, UK; 3 Research Institute of Sport and Exercise Science, Liverpool John Moores University, Liverpool, UK; 4 Clinical Trial Service Unit and Epidemiological Studies Unit, Nuffield Department of Population Health, University of Oxford, Oxford, UK

**Keywords:** Muscle mass, Mendelian randomization, 25-Hydroxyvitamin D, 25(OH)D, lean mass, fat-free mass

## Abstract

**Purpose:**

Low serum vitamin D status has been associated with reduced muscle mass in observational studies although the relationship is controversial and a causal association cannot be determined from such observations. Two-sample Mendelian randomization (MR) was applied to assess the association between serum vitamin D (25(OH)D) and total, trunk, arm and leg fat-free mass (FFM).

**Methods:**

MR was implemented using summary-level data from the largest genome-wide association studies (GWAS) on vitamin D (n=73,699) and total, trunk, arm and leg FFM. Inverse variance weighted method (IVW) was used to estimate the causal estimates. Weighted median (WM)-based method, and MR-Egger, leave-one-out were applied as sensitivity analysis.

**Results:**

Genetically higher serum 25(OH)D levels had a positive effect on total (IVW = Beta: 0.042, p = 0.038), trunk (IVW = Beta: 0.045, p = 0.023) and arm (right arm IVW = Beta: 0.044, p = 0.002; left arm IVW = Beta: 0.05, p = 0.005) FFM. However, the association with leg FFM was not significant (right leg IVW = Beta: 0.03, p = 0.238; left leg IVW = Beta: 0.039, p = 0.100). The likelihood of heterogeneity and pleiotropy was determined to be low (statistically non-significant), and the observed associations were not driven by single SNPs. Furthermore, MR pleiotropy residual sum and outlier test did not highlight any outliers.

**Conclusions:**

Our results illustrate the potentially causal, positive effect of serum 25(OH)D concentration on total, trunk and upper body appendicular fat-free mass.

## Introduction

**V**itamin D is an essential nutrient for human health with roles in multiple biological pathways and low vitamin D status is associated with multiple chronic diseases (1) as well as being associated with musculoskeletal health (2, 3) highlighting this nutrient's significance in the global burden of disease. However, up to 40% of the European population may suffer from vitamin D insufficiency (serum 25-hydroxy vitamin D [25(OH)D] concentration <50 nmol/L) (4) and vitamin D deficiency (25(OH)D concentration <30 nmol/L)is widespread enough to be considered a global health issue (4, 5, 6).

Loss of muscle mass directly affects muscle strength and physical function and as such, sarcopenia, the progressive loss of muscle mass and strength in aging, and frailty (7, 8). Furthermore, muscle mass loss has been associated with a multitude of chronic conditions including cardiovascular disease (CVD) (9), type 2 diabetes mellitus (T2DM) (10), increased risk of falls and fractures (11), cognitive decline and depression (12, 13), and all-cause mortality (14). Older adults may spend more time indoors due to poor mobility/reduced muscle function which can further lead to an elevated risk of vitamin D inadequacy (15, 16), leading to a vicious cycle of vitamin D deficiency and loss of muscle mass.

Epidemiological studies suggest an association between low vitamin D status and reduced muscle mass (3, 17, 18) although some studies have found no such association (19, 20). However, such studies are limited as observational data cannot determine whether an association is causal. Mendelian randomization (MR) analysis uses functional polymorphisms (single nucleotide polymorphisms (SNPs)) associated with specific changes in exposures (in this case, serum 25(OH) D) as genetic instruments to determine whether the risk factor is a cause of the disease (21). A major advantage of MR analysis is that they are considerably less prone to confounding, residual bias, and reverse causation than conventional risk-factor epidemiology (22). MR analysis may also circumvent the financial, logistical and ethical limitations of randomised controlled trials (RCTs) and additionally, the data from such studies can inform the design of pilot RCTs and clinical trials by providing information for the potential magnitude of effect of nutrients on a given outcome in specific populations (23).

In the present study, we used MR analysis to determine whether a potential causal relationship exists between serum 25(OH)D concentration and total, trunk, arm and leg fat-free mass (FFM).

## Methods

### Study design

A two-sample MR study design was used. In a 2-sample MR, the ssummary statistics are provided from various studies for the association of the genetic instruments with the exposure and outcome. In our study, we obtained the summary statistics from the largest genome wide association studies (GWAS) on serum 25(OH)D (exposure (24)) and FFM (outcome). We applied methods to estimate the unbiased effect of serum 25(OH)D on FFM (total, trunk, arms and legs,).

### Genetic predictors of exposures

We used six SNPs identified to be associated with circulating 25(OH)D concentration by the SUNLIGHT meta-GWAS, which are samples of European ancestry (79,366 discovery samples and 42,757 replication samples) (Table 1). GWAS were performed within each cohort according to a uniform analysis plan. Additive genetic models using linear regression on natural-log-transformed 25(OH)D were fitted and a fixed-effects inverse variance weighted (IVW) meta-analysis across the contributing cohorts was performed (24).

**Table 1:** Summary results of the 6 genetic loci associated with serum vitamin D

**SNP**	**Nearest gene**	**GX**	**GX SE**	**EA**	**OA**	**EAF**	**p-value**
rs3755967	GC	−0.089	0.0023	T	C	0.28	4.74E-343
rs10741657	CYP2R1	0.031	0.0022	A	G	0.4	2.05E-46
rs12785878	NADSYN1/DHCR7	0.036	0.0022	T	G	0.75	3.80E-62
rs10745742	AMDHD1	0.019	0.002	T	C	0.4	2.10E-20
rs8018720	SEC23A	−0.019	0.0027	C	G	0.82	1.11E-11
rs17216707	CYP24A1	0.026	0.0027	T	C	0.79	8.14E-23


All serum vitamin D markers were associated at genome-wide significance (p < 5 x 10^−8^); EA: effect allele; OA: other allele, EAF: effect allele frequency; GX: the per-allele effect on standard deviation units of the telomere length; GX SE: standard error of GX.

### Association of genetic instruments with outcome

SNPs associated with bioelectrical-impedance-measured fat mass and total, trunk, arm and leg FFM were obtained from analyses by Neale Lab (http://www.nealelab.is). We retrieved the association of the six genetic instruments with SNPs associated with bioelectrical impedance measured FFM using data obtained from UK Biobank. Detailed descriptions of the methods used to measure body composition is available on the UK Biobank website (25). Briefly, whole body as well as site-specific (trunk, leg, arm) fat-free mass/fat mass were evaluated with bioelectrical-impedance analysis (Tanita BC418MA body composition analyser). Body composition of a subset of participants was also assessed using dual-energy X-ray absorptiometry (DXA) which showed high correlation with bio-impedance values (fat-free mass: r = 0.96) (25). The UK Biobank is a population-based cohort of approximately 500,000 individuals; 54% are female, the average age is 57 (range 37–73), while 94% report as being White British. Further details on the rationale, design and methodology for UK Biobank can be found elsewhere (26).

### Mendelian Randomisation analysis

We combined the effect of six instruments using inverse variance weighted (IVW) method as implemented in Two Sample MR package of the statistical software, R (R Core Team, Vienna, Austria. https://www.R-project.org/). We assessed the heterogeneity using Q value for IVW. To address the potential effect of pleiotropic variants on the final effect estimate, we conducted sensitivity analysis including weighted median (WM) and MR-Egger. Sensitivity analysis was conducted using the leave-one-out method. The weighted median (WM) estimate, as the weighted median of the SNP-specific estimates, provides correct estimates as long as SNPs accounting for ≥50% of the weight are valid instruments. WM MR allows some variants to be invalid instruments provided at least half are valid instruments. It uses inverse variance weights and bootstrapping to estimate confidence intervals (CIs) (27). MR-Egger has an ability to make estimates by assumption of all SNPs are invalid instruments as long as the assumption of instrument strength independent of direct effect (InSIDE) is satisfied (27). MR-Egger allows free estimation of the intercept, although further assumptions, such as the independence between instrument strength and direct effects, cannot be easily verified. Average directional pleiotropy across genetic variants was assessed from the p-value of the intercept term from MR-Egger (27). Causal estimates in MR Egger are less precise than those obtained by using IVW MR (28). Analysis using MR-Egger has a lower false positive rate but a higher false negative rate than IVW (29).

Further, to assess heterogeneity between individual genetic variant estimates, we used the Q' heterogeneity statistic [30] and the MR pleiotropy residual sum and outlier (MR-PRESSO) test (30). The Q' statistic uses modified 2nd order weights that are a derivation of a Taylor series expansion and take into account uncertainty in both numerator and denominator of the instrumental variable ratio (this eases the no-measurement-error [NOME] assumption) (30). The MR-PRESSO framework relies on the regression of variant-outcome associations on variantexposure associations and implements a global heterogeneity test by comparing the observed distance (residual sums of squares) of all variants to the regression line with the distance expected under the null hypothesis of no pleiotropy (31). In case of evidence of horizontal pleiotropy, the test compares individual variants expected and observed distributions to identify outlier variants. Further we applied on MR-Robust Adjusted Profile Score (RAPS) this method is able to correct for pleiotropy using robust adjusted profile scores. We consider as results, causal estimates that agreed in direction and magnitude across MR methods, pass nominal significance in IVW MR, and did not show evidence of bias from horizontal pleiotropy using heterogeneity tests. We used R version 3.4.2 (R Core Development Team 2017).

The MR studies assume that the SNPs (instrumental variables) are associated with the outcome only via the exposure (32), so we performed sensitivity analysis excluding SNPs with potentially pleiotropic effects. To assess the instrumental variable analysis “exclusion-restriction” assumption we used Ensembl release (http://useast.ensembl.org/index.html). Ensembl contains a base of SNP phenotypes.

### Ethics

This investigation uses published or publicly available summary data with no involvement of participants in the study. No original data were collected for this manuscript. Ethical approval for each of the studies included in the investigation can be found in the original publications (including informed consent from each subject).

## Results

**Figure 1:**
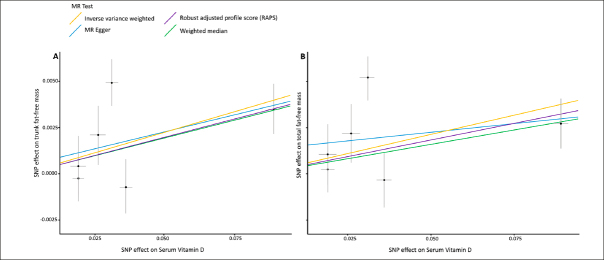
Scatter plots of the association of the effect of SNP-determined serum 25(OH)D on trunk (A) and total (B) fat-free mass

In total, 6 SNPs were identified as instrumental variables for serum 25(OH)D, none of which were significantly associated with FFM. A list of all SNP associations is shown in Table 1. The results of MR analysis, displayed as beta-coefficient for interested outcomes per increase in serum 25(OH) D, demonstrate a positive and statistically significant effect on total FFM (MR Egger= β:0.019, p= 0.657 and IVW=β: 0.042, p= 0.038; respectively, Table 2 and Fig. 1), trunk (MR Egger= β:0.037, p= 0.406 and IVW=β: 0.045, p=0.023, respectively, Table 2 and Fig. 1) FFM. This data suggests that each 25 nmol/L increase in serum 25(OH)D is associated with an increase of 0.042 kg of total FFM. Serum 25(OH) D also demonstrated a positive and statistically significant effect on arm FFM (Right arm: MR Egger= β:0.043, p= 0.225 and IVW=β: 0.044, p=0.002; Left arm: MR Egger= β:0.033, p= 0.398 and IVW=β: 0.05, p=0.005, respectively, Table 2. However, results for leg FFM did not demonstrate a statistically significant effect (Right leg: MR Egger= β: -0.025, SE: 0.04, p= 0.561 and IVW=β: 0.03, SE: 0.026, p=0.238; Left leg: MR Egger= β: −0.008, SE: 0.038, p= 0.838 and IVW=β: 0.039, SE: 0.023, p=0.1, respectively, Table 2).

**Table 2:** Results of the Mendelian Randomization (MR) analysis for effects of serum vitamin D on total, trunk, arm and leg fat-free mass

**Exposure**	**Outcome**	**MR**				**Heterogeneity**			**Pleiotropy**		
		**Method**	**beta**	**SE**	**p**	**Method**	**Q**	**P-value**	**Intercept**	**SE**	**p**
Vitamin D (Serum 25(OH)D)	Total fat-free mass	MR Egger	0.019	0.039	0.657	MR-Egger	11.018	0.026	0.001	0.002	0.503
		WM	0.031	0.015	0.029						
		IVW	0.042	0.02	0.038	IVW	12.506	0.029			
		RAPS	0.036	0.016	0.03						
	Trunk fat-free mass	MR Egger	0.037	0.039	0.406	MR-Egger	11.479	0.022	0.0004	0.002	0.817
		WM	0.039	0.015	0.008						
		IVW	0.045	0.02	0.023	IVW	11.655	0.04			
		RAPS	0.039	0.017	0.019						
	Arm fat-free mass (right)	MR Egger	0.042	0.029	0.225	MR-Egger	6.415	0.17	0.0001	0.001	0.94
		WM	0.042	0.014	0.003						
		IVW	0.044	0.015	0.002	IVW	6.425	0.267			
		RAPS	0.042	0.014	0.002						
	Arm fat-free mass (left)	MR Egger	0.033	0.0349	0.398	MR-Egger	8.746	0.068	0.001	0.002	0.589
		WM	0.041	0.015	0.007						
		IVW	0.05	0.018	0.005	IVW	9.499	0.091			
		RAPS	0.046	0.016	0.005						
	Leg fat-free mass (right)	MR Egger	−0.025	0.0401	0.561	MR-Egger	10.775	0.029	0.003	0.002	0.171
		WM	0.015	0.0152	0.334						
		IVW	0.03	0.0258	0.238	IVW	18.269	0.003			
		RAPS	0.022	0.021	0.281						
	Leg fat-free mass (left)	MR Egger	−0.008	0.0382	0.838	MR-Egger	9.807	0.044	0.003	0.002	0.215
		WM	0.022	0.0148	0.143						
		IVW	0.039	0.0234	0.1	IVW	15.108	0.009			
		RAPS	0.031	0.019	0.098						


25(OH)D: 25-hydroxy vitamin D; WM: weighted median; IVW: inverse variance weighted; SE: standard error; beta: beta-coefficients; MR: Mendelian randomization; RAPS: robust adjusted profile score

Each black point represents an SNP, plotted by the estimate of SNP on serum 25(OH)D level (x-axis, nmol/L) and the estimate of SNP on fat-free mass (y-axis, kg). The slopes of each line represent the potential causal associations for each method.

The horizontal pleiotropy test, with very negligible Egger regression intercept, also showed a low likelihood of pleiotropy for all our estimations (all p > 0.171, Table 2). Further the result of the MR-RAPS was identical with the IVW prediction, which again indicated a statistically low chance of pleiotropy. Heterogeneity tests highlighted no trace of heterogeneity (Table 2). Furthermore, MR-PRESSO analysis did not indicate any outliers for all estimates. Results of leave-one-out method demonstrated that the links are not driven by any single SNP.

## Discussion

Our results illustrate the potentially causal, positive effect of lifetime serum 25(OH)D concentration on total, trunk and arm FFM. These findings are in agreement with a number of cross-sectional, population-based studies, which have shown a positive relationship between serum 25(OH)D status and FFM in a wide range of age groups and clinical populations [3, 17, 18]. In a study of 100 adolescents (15.1 ± 1.9 y), serum 25(OH)D was positively associated with lean body mass and inversely with fat mass (18). In a cross-sectional study of 127 pre-frail and frail elderly people (79.0 ± 7.8 y) in The Netherlands, Tieland et al. (3) reported that low 25(OH)D status was associated with reduced muscle mass and poorer physical performance (3). Additionally, a meta-analysis of 12 studies with data from 22,590 individuals (mean range 50 – 88 yrs) reported that sarcopenic individuals had lower blood 25(OH)D concentrations than non-sarcopenic controls (17). Conversely, some studies have reported no such association between 25(OH)D and lean body mass (LBM) or FFM (19, 20).

Mechanistically, vitamin D is known to exert its effects of muscle tissue both by regulating expression of target genes via the vitamin D receptor (VDR) and by non-genomic regulation of skeletal muscle intracellular signaling pathways (33). In animal models, vitamin D supplementation has been demonstrated to activate the mammalian target of rapamycin/S6 kinase (mTOR/S6K) pathway, which leads to increased muscle protein synthesis (MPS) (34) essential for increases in muscle protein accrual and size (35). Cell culture models have also reported that vitamin D enhances the stimulating effect of leucine and insulin on muscle protein synthesis rates (36) and promotes myogenic differentiation and reduces the expression of myostatin, a known negative regulator of muscle size (37). Vitamin D has also been reported to stimulate the expression of genes involved in the control of cellular growth (33, 38). These varied mechanisms may partly explain the adverse effects of low vitamin D status on muscle mass and function.

The present study did not find a statistically significant relationship between genetically determined serum 25(OH)D concentration and leg FFM. This is not the first study to identify a discrepancy in the relationship between 25(OH)D status with upper and lower body appendicular lean mass. In a study of frail elderly Dutch people (n = 127; mean 79 y) 25(OH)D status was associated with appendicular lean mass (ALM) (β=0.012 [P=0.05]) but was not significantly associated with leg lean mass (β=0.008 [P=0.08]) (3). Furthermore, a cross-sectional study of the association of 25(OH)D status with muscle strength (n = 419; healthy men and women; 20–76 y) has also reported a stronger association between 25(OH)D and muscle strength in the arms compared to the legs (39). One potential explanation for this discrepancy is the reported greater distribution of VDR in type 2 muscle fibres (40) which make up a greater proportion of upper body skeletal muscle (41, 42, 43, 44). Vitamin D affects both the diameter and the number of type 2 muscle fibres, which are important for not only young athletes but also the elderly, due to their capacity to reduce the risk of falls, for example (45, 46). Greater expression of VDR has been reported to stimulate muscle hypertrophy through a number of potential mechanisms including increased protein synthesis (47). Furthermore, the greater daily utilization of lower extremities, for example, due to locomotion and bearing the individuals body weight during movement, may provide a superior stimulus for muscle hypertrophy. Further research is clearly needed to elucidate the mechanisms by which vitamin D differentially affects lower and upper body appendicular muscle physiology.

This study highlights the importance of serum vitamin D concentrations in accruing and maintaining FFM, which itself is associated with lower risk of frailty and mortality (7, 11, 14). Addressing vitamin D insufficiency is challenging as the main source of vitamin D in humans is sun exposure (48) which is unlikely to become a widely accepted and implemented strategy. Furthermore, dietary intakes of vitamin D are typically low (49) due to low levels in common foodstuffs (50). Therefore, at a population level, food fortification with vitamin D, and at an individual level, supplementation may be the most effective methods to increase 25(OH)D status to sufficient levels (5).

A major strength of our study was the large sample population study with access to individual participant data of high validity and with the relevant SNPs available for both 25(OH)D serum concentration and FFM. Furthermore, the use of the Mendelian randomisation approach allowed us to examine the potential causal effects of serum 25(OH)D, largely without the disadvantages of confounding or reverse causation.

A potential limitation of this study is the use of segmental bioelectrical impedance analysis (BIA) as the method for determining FFM in the UK Biobank cohort. The accuracy of BIA measurement is known to be affected by hydration status; however the UK Biobank protocol did not specify any procedures to standardise some determinants of hydration before the assessment. This could potentially lead to inaccuracies in the values attained for FFM (51). Furthermore, evidence suggests that BIA is less accurate at high BMI levels (52) and considering the range of BMI included in the UK Biobank cohort, this should be taken into consideration with these results.

## Conclusions

Evidence for a potentially causal association of serum 25(OH)D with total, trunk and arm FFM was found. However, the relationship between serum 25(OH)D and leg FFM was not statistically significant. This finding highlights the importance of maintaining sufficient 25(OH)D status throughout the life course in order to maintain adequate lean mass, a factor associated with multiple chronic disease. Future research should address the causal role and potential mechanisms of serum 25(OH)D on FFM accrual and maintenance as well as the apparent lack of effect on leg FFM.

## References

[1] Wang H, Chen W, Li D, Yin X, Zhang X, Olsen N, Zheng SG (2017). Vitamin D and Chronic Diseases. Aging Dis.

[2] Girgis CM (2020). Vitamin D and Skeletal Muscle: Emerging Roles in Development, Anabolism and Repair. Calcif Tissue Int.

[3] Tieland M, Brouwer-Brolsma EM, Nienaber-Rousseau C, van Loon LJC, De Groot LCPGM (2013). Low vitamin D status is associated with reduced muscle mass and impaired physical performance in frail elderly people. European Journal of Clinical Nutrition.

[4] Cashman KD, Dowling KG, Škrabáková Z, Gonzalez-Gross M, Valtueña J, De Henauw S, Moreno L, Damsgaard CT, Michaelsen KF, Mølgaard C, Jorde R, Grimnes G, Moschonis G, Mavrogianni C, Manios Y, Thamm M, Mensink GB, Rabenberg M, Busch MA, Cox L, Meadows S, Goldberg G, Prentice A, Dekker JM, Nijpels G, Pilz S, Swart KM, van Schoor NM, Lips P, Eiriksdottir G, Gudnason V, Cotch MF, Koskinen S, Lamberg-Allardt C, Durazo-Arvizu RA, Sempos CT, Kiely M (2016). Vitamin D deficiency in Europe: pandemic?. Am J Clin Nutr.

[5] Cashman KD (2020). Vitamin D Deficiency: Defining, Prevalence, Causes, and Strategies of Addressing. Calcif Tissue Int.

[6] Del Valle HB, Yaktine AL, Taylor CL, Ross AC. Dietary reference intakes for calcium and vitamin D. 2011. 21796828

[7] Xu W, Chen T, Cai Y, Hu Y, Fan L, Wu C (2020). Sarcopenia in Community-Dwelling Oldest Old is Associated with Disability and Poor Physical Function. The journal of nutrition, health & aging.

[8] Cruz-Jentoft AJ, Bahat G, Bauer J, Boirie Y, Bruyere O, Cederholm T, Cooper C, Landi F, Rolland Y, Sayer AA, Schneider SM, Sieber CC, Topinkova E, Vandewoude M, Visser M, Zamboni M (2019). Sarcopenia: revised European consensus on definition and diagnosis. Age Ageing.

[9] Bahat G, İlhan B (2016). Sarcopenia and the cardiometabolic syndrome: A narrative review. European Geriatric Medicine.

[10] Scott D, de Courten B, Ebeling PR (2016). Sarcopenia: a potential cause and consequence of type 2 diabetes in Australia’s ageing population?. The Medical journal of Australia.

[11] Schaap LA, van Schoor NM, Lips P, Visser M (2018). Associations of Sarcopenia Definitions, and Their Components, With the Incidence of Recurrent Falling and Fractures: The Longitudinal Aging Study Amsterdam. J Gerontol A Biol Sci Med Sci.

[12] Hayashi T, Umegaki H, Makino T, Cheng XW, Shimada H, Kuzuya M (2019). Association between sarcopenia and depressive mood in urban-dwelling older adults: A cross-sectional study. Geriatr Gerontol Int.

[13] Hsu YH, Liang CK, Chou MY, Liao MC, Lin YT, Chen LK, Lo YK (2014). Association of cognitive impairment, depressive symptoms and sarcopenia among healthy older men in the veterans retirement community in southern Taiwan: a cross-sectional study. Geriatr Gerontol Int.

[14] Abramowitz MK, Hall CB, Amodu A, Sharma D, Androga L, Hawkins M (2018). Muscle mass, BMI, and mortality among adults in the United States: A population-based cohort study. PLoS One.

[15] Webb AR, Pilbeam C, Hanafin N, Holick MF (1990). An evaluation of the relative contributions of exposure to sunlight and of diet to the circulating concentrations of 25-hydroxyvitamin D in an elderly nursing home population in Boston. Am J Clin Nutr.

[16] Whitmore SE (1996). Vitamin D deficiency in homebound elderly persons. Jama.

[17] Luo J, Quan Z, Lin S, Cui L (2018). The association between blood concentration of 25-hydroxyvitamin D and sarcopenia: a meta-analysis. Asia Pac J Clin Nutr.

[18] Wierzbicka E, Szalecki M, Pludowski P, Jaworski M, Brzozowska A (2016). Vitamin D status, body composition and glycemic control in Polish adolescents with type 1 diabetes. Minerva Endocrinol.

[19] Ceglia L, Chiu GR, Harris SS, Araujo AB (2011). Serum 25-hydroxyvitamin D concentration and physical function in adult men. Clinical Endocrinology.

[20] De Pergola G, Martino T, Zupo R, Caccavo D, Pecorella C, Paradiso S, Silvestris F, Triggiani V (2019). 25 Hydroxyvitamin D Levels are Negatively and Independently Associated with Fat Mass in a Cohort of Healthy Overweight and Obese Subjects. Endocr Metab Immune Disord Drug Targets.

[21] Larsson SC (2021). Mendelian randomization as a tool for causal inference in human nutrition and metabolism. Current opinion in lipidology.

[22] Smith GD, Ebrahim S (2003). ‘Mendelian randomization’: can genetic epidemiology contribute to understanding environmental determinants of disease?. Int J Epidemiol.

[23] Plotnikov D, Guggenheim JA (2019). Mendelian randomisation and the goal of inferring causation from observational studies in the vision sciences. Ophthalmic and Physiological Optics.

[24] Jiang X, O’Reilly PF, Aschard H, Hsu YH, Richards JB, Dupuis J, Ingelsson E, Karasik D, Pilz S, Berry D, Kestenbaum B, Zheng J, Luan J, Sofianopoulou E, Streeten EA, Albanes D, Lutsey PL, Yao L, Tang W, Econs MJ, Wallaschofski H, Volzke H, Zhou A, Power C, McCarthy MI, Michos ED, Boerwinkle E, Weinstein SJ, Freedman ND, Huang WY, Van Schoor NM, van der Velde N, Groot L, Enneman A, Cupples LA, Booth SL, Vasan RS, Liu CT, Zhou Y, Ripatti S, Ohlsson C, Vandenput L, Lorentzon M, Eriksson JG, Shea MK, Houston DK, Kritchevsky SB, Liu Y, Lohman KK, Ferrucci L, Peacock M, Gieger C, Beekman M, Slagboom E, Deelen J, Heemst DV, Kleber ME, Marz W, de Boer IH, Wood AC, Rotter JI, Rich SS, Robinson-Cohen C, den Heijer M, Jarvelin MR, Cavadino A, Joshi PK, Wilson JF, Hayward C, Lind L, Michaelsson K, Trompet S, Zillikens MC, Uitterlinden AG, Rivadeneira F, Broer L, Zgaga L, Campbell H, Theodoratou E, Farrington SM, Timofeeva M, Dunlop MG, Valdes AM, Tikkanen E, Lehtimaki T, Lyytikainen LP, Kahonen M, Raitakari OT, Mikkila V, Ikram MA, Sattar N, Jukema JW, Wareham NJ, Langenberg C, Forouhi NG, Gundersen TE, Khaw KT, Butterworth AS, Danesh J, Spector T, Wang TJ, Hypponen E, Kraft P, Kiel DP (2018). Genome-wide association study in 79,366 European-ancestry individuals informs the genetic architecture of 25-hydroxyvitamin D levels. Nature communications.

[25] UK Biobank-Body Composition Measurement. 2011. https://biobank.ctsu.ox.ac.uk/showcase/showcase/docs/body_composition.pdf. Accessed August 15, 2021.

[26] Sudlow C, Gallacher J, Allen N, Beral V, Burton P, Danesh J, Downey P, Elliott P, Green J, Landray M (2015). UK biobank: an open access resource for identifying the causes of a wide range of complex diseases of middle and old age. PLoS medicine.

[27] Bowden J, Davey Smith G, Haycock PC, Burgess S (2016). Consistent Estimation in Mendelian Randomization with Some Invalid Instruments Using a Weighted Median Estimator. Genetic epidemiology.

[28] Bowden J, Davey Smith G, Burgess S (2015). Mendelian randomization with invalid instruments: effect estimation and bias detection through Egger regression. International journal of epidemiology.

[29] Burgess S, Bowden J, Fall T, Ingelsson E, Thompson SG (2017). Sensitivity Analyses for Robust Causal Inference from Mendelian Randomization Analyses with Multiple Genetic Variants. Epidemiology (Cambridge, Mass).

[30] Bowden J, Del Greco MF, Minelli C, Davey Smith G, Sheehan N, Thompson J (2017). A framework for the investigation of pleiotropy in two-sample summary data Mendelian randomization. Statistics in medicine.

[31] Verbanck M, Chen CY, Neale B, Do R (2018). Detection of widespread horizontal pleiotropy in causal relationships inferred from Mendelian randomization between complex traits and diseases. Nature genetics.

[32] Lawlor DA, Harbord RM, Sterne JA, Timpson N, Davey Smith G (2008). Mendelian randomization: using genes as instruments for making causal inferences in epidemiology. Statistics in medicine.

[33] Boland RL (2011). VDR activation of intracellular signaling pathways in skeletal muscle. Mol Cell Endocrinol.

[34] Vignale K, Greene ES, Caldas JV, England JA, Boonsinchai N, Sodsee P, Pollock ED, Dridi S, Coon CN (2015). 25-Hydroxycholecalciferol Enhances Male Broiler Breast Meat Yield through the mTOR Pathway. The Journal of nutrition.

[35] Atherton PJ, Smith K (2012). Muscle protein synthesis in response to nutrition and exercise. The Journal of physiology.

[36] Salles J, Chanet A, Giraudet C, Patrac V, Pierre P, Jourdan M, Luiking YC, Verlaan S, Migné C, Boirie Y, Walrand S (2013). 1,25(OH)2-vitamin D3 enhances the stimulating effect of leucine and insulin on protein synthesis rate through Akt/PKB and mTOR mediated pathways in murine C2C12 skeletal myotubes. Mol Nutr Food Res.

[37] Garcia LA, King KK, Ferrini MG, Norris KC, Artaza JN (2011). 1,25(OH)2vitamin D3 stimulates myogenic differentiation by inhibiting cell proliferation and modulating the expression of promyogenic growth factors and myostatin in C2C12 skeletal muscle cells. Endocrinology.

[38] Neary J (1997). MAPK Cascades in Cell Growth and Death. Physiology.

[39] Grimaldi AS, Parker BA, Capizzi JA, Clarkson PM, Pescatello LS, White MC, Thompson PD (2013). 25(OH) vitamin D is associated with greater muscle strength in healthy men and women. Medicine and science in sports and exercise.

[40] Srikuea R, Hirunsai M, Charoenphandhu N (2020). Regulation of vitamin D system in skeletal muscle and resident myogenic stem cell during development, maturation, and ageing. Sci Rep.

[41] Ørtenblad N, Nielsen J, Boushel R, Söderlund K, Saltin B, Holmberg HC (2018). The Muscle Fiber Profiles, Mitochondrial Content, and Enzyme Activities of the Exceptionally Well-Trained Arm and Leg Muscles of Elite Cross-Country Skiers. Front Physiol.

[42] Johnson MA, Polgar J, Weightman D, Appleton D (1973). Data on the distribution of fibre types in thirty-six human muscles. An autopsy study. J Neurol Sci.

[43] Klein CS, Marsh GD, Petrella RJ, Rice CL (2003). Muscle fiber number in the biceps brachii muscle of young and old men. Muscle & nerve.

[44] Travnik L, Pernus F, Erzen I (1995). Histochemical and morphometric characteristics of the normal human vastus medialis longus and vastus medialis obliquus muscles. J Anat.

[45] (2019). Nutrients.

[46] Koundourakis NE, Avgoustinaki PD, Malliaraki N, Margioris AN (2016). Muscular effects of vitamin D in young athletes and non-athletes and in the elderly. Hormones (Athens).

[47] Bass JJ, Nakhuda A, Deane CS, Brook MS, Wilkinson DJ, Phillips BE, Philp A, Tarum J, Kadi F, Andersen D, Garcia AM, Smith K, Gallagher IJ, Szewczyk NJ, Cleasby ME, Atherton PJ (2020). Overexpression of the vitamin D receptor (VDR) induces skeletal muscle hypertrophy. Mol Metab.

[48] Engelsen O (2010). The Relationship between Ultraviolet Radiation Exposure and Vitamin D Status. Nutrients.

[49] Kiely M, Black LJ (2012). Dietary strategies to maintain adequacy of circulating 25-hydroxyvitamin D concentrations. Scand J Clin Lab Invest Suppl.

[50] Schmid A, Walther B (2013). Natural Vitamin D Content in Animal Products. Advances in Nutrition.

[51] Kyle UG, Bosaeus I, De Lorenzo AD, Deurenberg P, Elia M, Manuel Gómez J, Lilienthal Heitmann B, Kent-Smith L, Melchior JC, Pirlich M, Scharfetter H, AMWJS, Pichard C (2004). Bioelectrical impedance analysis-part II: utilization in clinical practice. Clinical nutrition (Edinburgh, Scotland).

[52] Neovius M, Hemmingsson E, Freyschuss B, Uddén J (2006). Bioelectrical impedance underestimates total and truncal fatness in abdominally obese women. Obesity (Silver Spring).

